# Poultry health constraints in smallholder village poultry systems in Northern Ghana and Central Tanzania

**DOI:** 10.3389/fvets.2023.1159331

**Published:** 2023-07-03

**Authors:** Emily Awuor Ouma, Clovice Kankya, Michel Dione, Terra Kelly, Dolapo Enahoro, Gaspar Chiwanga, Yakubu Abukari, Peter Msoffe, Boniface Baboreka Kayang, Huaijun Zhou

**Affiliations:** ^1^International Livestock Research Institute, Nairobi, Kenya; ^2^Feed the Future Innovation Lab for Genomics to Improve Poultry, University of California, Davis, Davis, CA, United States; ^3^Department of Biosecurity, Ecosystems and Veterinary Public Health, College of Veterinary Medicine, Animal Resources and Biosecurity, Makerere University, Kampala, Uganda; ^4^One Health Institute, University of California, Davis, Davis, CA, United States; ^5^Tanzania Veterinary Laboratory Agency, South Zone, Mtwara, Tanzania; ^6^Regional Department of Agriculture, Northern Regional Coordinating Council, Tamale, Ghana; ^7^Department of Veterinary Medicine and Public Health, Sokoine University of Agriculture, Morogoro, Tanzania; ^8^Department of Animal Science, School of Agriculture, College of Basic and Applied Sciences, University of Ghana, Accra, Ghana; ^9^Department of Animal Science, University of California, Davis, Davis, CA, United States

**Keywords:** village poultry systems, local chicken ecotypes, participatory epidemiology, Newcastle disease, Ghana, Tanzania

## Abstract

**Introduction:**

Smallholder poultry production is a major contributor to food security and rural livelihoods in low-and middle-income countries. However, infectious diseases limit improvements to smallholder poultry production and performance of the sector in general. Infectious diseases of poultry, especially viral diseases, have major impacts on the health and productivity of flocks and account for significant morbidities and mortalities of birds each year.

**Methods:**

This study utilized participatory epidemiology approaches to better understand the poultry health constraints and challenges faced by smallholder poultry producers in village poultry systems in Northern Ghana and Central Tanzania.

**Results:**

The results show dominance of small-scale semi-intensive and extensive scavenging poultry production systems in the study areas. Newcastle disease ranked as the highest cause of morbidity and mortality in chickens in the two countries. The disease occurred mainly during the months coinciding with the dry season in both countries. Other health challenges among poultry flocks included worm infestation, fowl pox, coryza, and coccidiosis. Producers, especially in rural locations, had poor access to veterinary services and critical inputs necessary for poultry production. In the Northern region of Ghana, producers lacked definitive diagnoses for sick poultry due to a shortage of veterinary personnel and diagnostic laboratories.

**Discussion:**

These challenges point to the need for increased investment in poultry disease control and prevention programs, particularly in rural areas. Interventions focused on expansion of veterinary and agricultural extension services and diagnostic laboratory capacity in rural areas and increased gender-sensitive training to enhance smallholder knowledge in poultry husbandry and disease prevention measures will support the development of the smallholder village poultry systems. Tapping into the diverse genetic reservoir of local chicken ecotypes with enhanced resistance to Newcastle disease through genomic selection, coupled with models for enhancing ND vaccination supply and use in the rural areas are potential future avenues for addressing ND constraints to production.

## Introduction

Smallholder poultry production is a promising venture that not only supports food security and nutrition but also addresses poverty related challenges in livelihood development in low-and middle-income countries (LMICs). In addition, poultry production is important for income generation and poverty alleviation, especially for women in rural households, as chickens can be sold or bartered to meet essential household needs (1).

Ghana and Tanzania are LMICs geographically situated within the Sub-Saharan Africa region (SSA). In both countries, the poultry sector is a key livelihood activity for both urban and rural populations. The countries have a comparable population growth rate of about 2% *per annum*—that is, 2.2 and 2.9% for Ghana and Tanzania, respectively (2). Such exponential human population growth increases demand for food and nutrients for growth and development. Therefore, this puts the poultry sector at a pivotal point to meet the increasing demand for animal source protein and to support livelihood development.

In Tanzania and Ghana, the poultry production sector is primarily characterized by local indigenous chickens and a smaller proportion of dual-purpose cross breeds (3). In addition, many poultry producers raise their chicken in mixed flocks with other birds, such as guinea fowl, pigeons, doves, turkeys, and ducks. The populations of indigenous chickens in Ghana and Tanzania are estimated at 38 million (4) and 43 million (5), respectively. In Tanzania, the bulk of the indigenous chicken population is reared in Central Tanzania, especially in the Singida, Mbeya, Tabora, and Morogoro regions. In Ghana, most of the population of indigenous chickens (about 50%) are reared in the Northern and Upper East regions.

In both countries, indigenous chicken populations are mainly raised under the extensive scavenging system with smallholders rearing about 20–50 birds per household. This system is a relatively low-input system requiring minimal feed supplementation for the flocks. There is high potential for growth in the production of local indigenous chicken ecotypes and dual-purpose breeds due to the increase in demand and preference by consumers for their meat and eggs (6, 7). This is a result of the growth in the middle-income class and an increase in disposable income in the urban areas (8).

While local poultry production holds great potential for improvements to household livelihoods and nutritional security, it is constrained by losses due to periodic disease outbreaks and by low productivity, especially during the dry season when poultry feed items are scarce (9). Disease outbreaks account for substantial morbidity and mortality of birds in village poultry systems where implementation of biosecurity measures is a challenge as chickens free-range and comingle with other domestic fowl and wild birds, which can serve as sources of infection. Vaccination programs can also be difficult to implement in such systems due to limited veterinary extension services, challenges in maintaining a cold chain to keep vaccines viable, and unreliable vaccine production and distribution in resource limited settings.

In Ghana and Tanzania, Newcastle disease (NCD) is a leading cause of mortality (70–100%) and morbidity (~80%) in poultry production (10, 11). There are ongoing efforts to enhance resistance to NCD in indigenous poultry ecotypes in Ghana and Tanzania through genomic selection as a complementary approach to NCD vaccination (12). Though NCD is identified as a critical constraint in most village poultry systems, it is important to understand the underlying factors and specific health constraints to inform on more holistic interventions. This study examines poultry health constraints in village poultry systems in Northern Ghana and Central Tanzania, to inform on potential interventions.

## Materials and methods

### Study area

**Figure 1:**
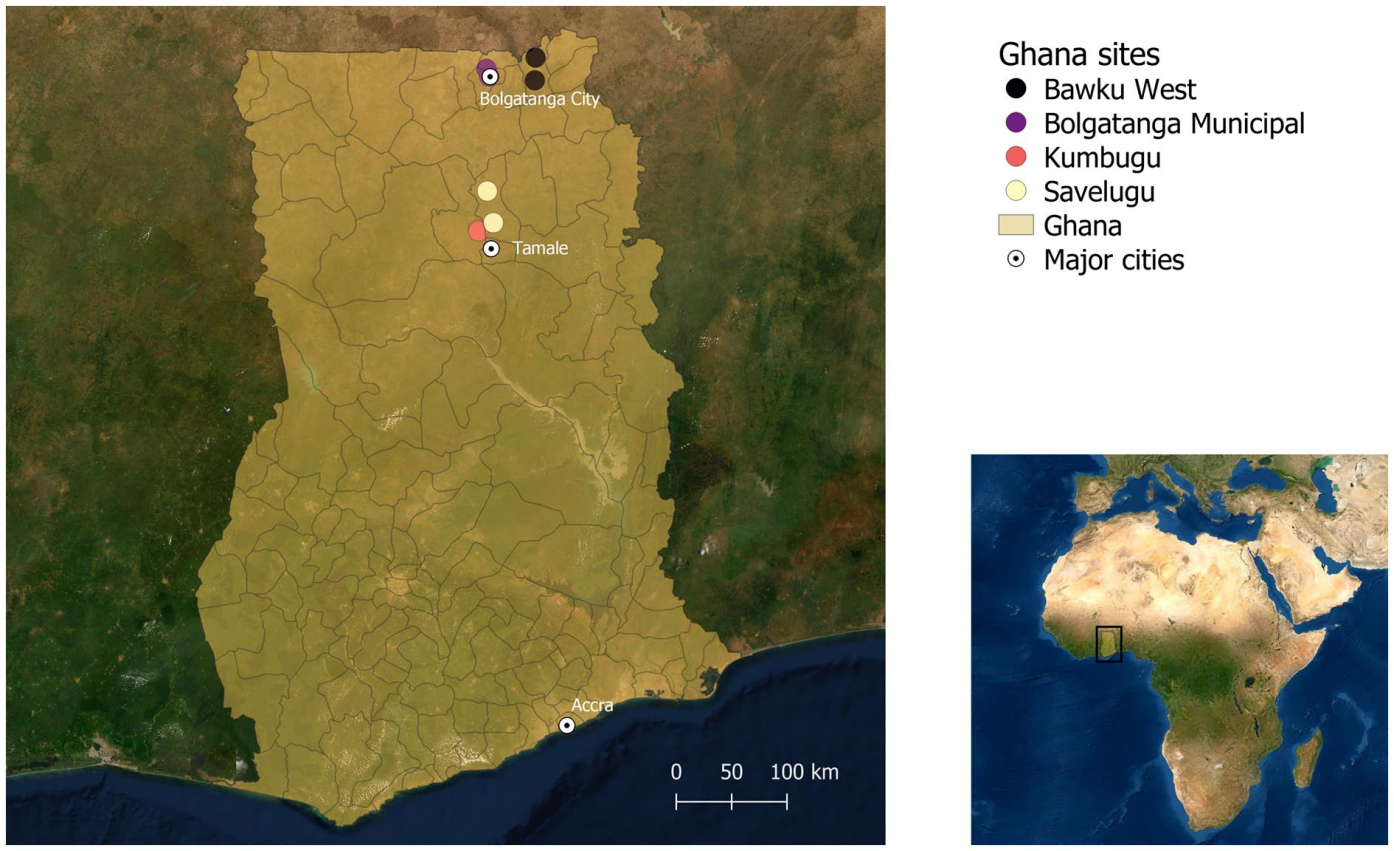
Study sites in Ghana (13).

**Figure 2:**
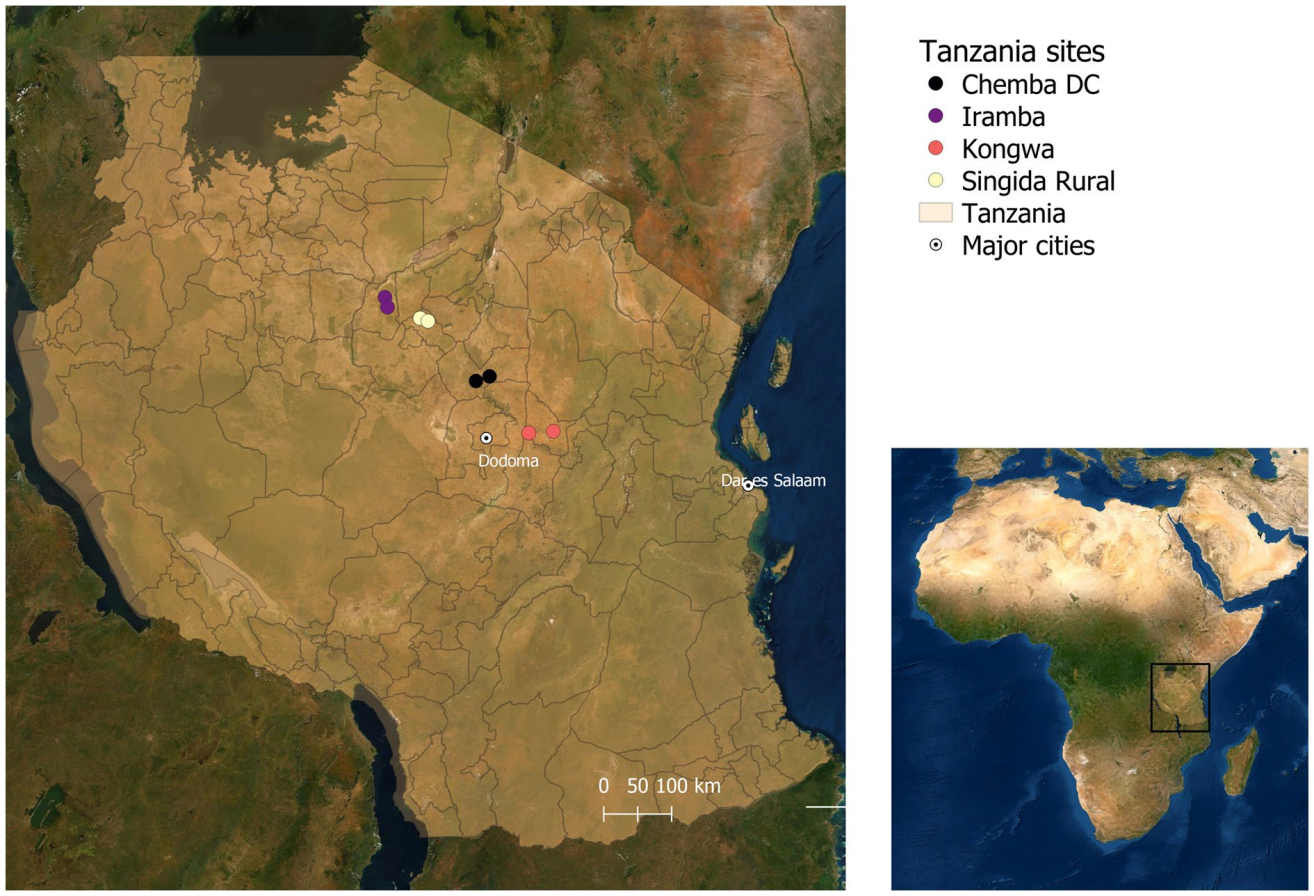
Study sites in Tanzania (13).

This study was conducted between November 2019 and March 2020 in the Upper East and Northern regions in Northern Ghana and Singida and Dodoma regions in Central Tanzania. It was part of a broader research project implemented in Ghana and Tanzania focusing on genetic selection for enhanced resistance to NCD and improved productivity in indigenous chicken ecotypes for breeding and distribution to smallholder farmers.^1^ In each region, two districts were selected for the assessments of poultry health challenges. The main selection criteria for the districts were large populations of smallholder poultry producers rearing chickens in extensive scavenging systems, proximity to demand areas for indigenous chicken ecotypes—mainly large urban areas, and incidence of NCD reported by the veterinary officers (8, 13). Within each district, two second level administrative divisions were selected (Metropolitan, Municipal and District Assembly, or MMDA for Ghana and Ward for Tanzania), with one site representing peri-urban chicken production and the other rural chicken production, far from urban demand centers (Table 1). This yielded a total of eight sites per country as depicted in the spatial maps in Figure 1 for Ghana and Figure 2 for Tanzania.

**Table 1:** Study sites.

Country	Region	District/Municipality	Study sites
Ghana	Upper East	Bolgatanga	Nyariga
			Sherigu
		Bawku West	Zebila
			Kukore
	Northern	Kumbugu	Kumbugu
			Gbulung
		Savelugu	Diare
			Savelugu
Tanzania	Dodoma	Kongwa	Mbande/Sejeli
			Kibaigwa
		Chemba	Gwandi
			Farkwa
	Singida	Iramba DC	Old Kiomboi
			Ulemo
		Singida rural	Ikhanoda
			Mtinko

The selection of the second level administrative divisions was performed by community-based focal persons and local leaders, with guidance from the research team.

### Sample size and survey implementation

The poultry producers who participated in the focus group discussions (FGDs) were randomly drawn from lists generated by the village chiefs in collaboration with agricultural extension staff at each study site as previously described by Enahoro et al. (13). For each site, one mixed-gender FGD comprising 12–15 farmers was conducted. A total of 16 FGDs (eight FGDs per country, one FGD for each study site) with 260 participants were conducted comprising 129 men and 131 women poultry producers. For each site, a local veterinary extension worker was invited to participate in the FGDs to provide more background on the poultry diseases and to translate the local names of the diseases indicated by the participants. Each FGD lasted a maximum of 2 h and was implemented by a facilitator and a note-taker who were recruited from the study regions and spoke the dialects of the communities. For each session, the facilitator facilitated the discussions in the local dialect and the note-taker took notes and recorded discussion proceedings. All facilitators were trained by experienced project scientists prior to the FGDs.

### Data collection

We utilized participatory epidemiology tools to enable identification and prioritization of poultry health challenges by the local communities. Participatory epidemiology (PE) approaches, which included pairwise ranking, proportional piling, and seasonal calendars, were used to collect data through FGDs with the smallholder producers at each site (14). The FGDs were guided by a semi-structured checklist that focused on poultry management systems and current husbandry practices, chicken flock entry and exit, disease prioritization and its impact on poultry production, as well as disease control options. The discussions also incorporated a seasonal calendar to understand temporal patterns of disease occurrence and main constraints to chicken production. Morbidity, mortality, and case fatality ratios were also calculated based on the flock data collected. The PE tools were applied with a lot of flexibility according to the local context to enable collection of authentic data. The detailed description of application of the specific PE tools follows below.

#### Simple and pairwise ranking

Simple ranking was applied to obtain a list of common poultry husbandry practices, common poultry diseases, and key constraints and challenges encountered by the smallholder poultry producers. For each aspect, the issues were listed on a card and participants were asked to organize or rank the cards in order of importance of the issue. For pairwise ranking, each listed item was compared individually with all other items one by one to investigate the alternative in the pair that is higher or equally ranked. This method was useful when there was a long list of items to be ranked, or the participants could not reach consensus using the simple ranking method or when two or more items had the same scores or rank through simple ranking (14). Pairwise ranking was used to prioritize diseases as well as constraints to chicken production.

#### Proportional piling

Proportional piling was used to investigate the relative contribution of various poultry diseases and health challenges to morbidity and mortality among the producers’ poultry flocks (14). It was also used to assess the sources of chicken entries and exits from the flock. Circles were drawn on paper with each circle representing a poultry health challenge or disease prioritized by the community. Participants were then provided 100 counters to represent the poultry population in their community making up 100% of the flock. The magnitude of the poultry disease and health challenge in terms of the proportion of birds affected was then identified by the number of counters in each circle as decided by the participants through consensus. The morbidity rate due to the disease was then calculated as the number of counters associated with the disease divided by the population (total counters). Further, the number of birds that were confirmed or suspected to have died due to the listed disease was also reflected by the number of counters the participants placed on circles representing death due to the disease or challenge. The mortality rate was then calculated as the number of birds that died due to the disease divided by the population (total counters). The case fatality ratio was calculated as the number of counters representing dead birds due to the disease divided by counters representing sick birds due to the disease. The same approach was used to probe participants on the proportions of their birds entering and exiting the flock through different mechanisms (e.g., hatching, purchase, death, and sales).

#### Seasonal calendar

A seasonal calendar was used to assess the occurrence of the prioritized poultry diseases across months in a calendar year for the different study locations (14). Participants drew 12 columns on a piece of paper with each column representing a month of the year (January to December). The list of prioritized diseases identified from the simple and pairwise ranking was listed on the first column before the calendar months. For each disease, participants were requested to place the counters in cells representing months of the year that through their experience, they perceived the disease to occur in their flocks. The number of counters placed against each month represented occurrence of that specific disease within the month.

### Data management and analysis

The data on FGD participant demographic characteristics, poultry husbandry practices, and production systems, factors associated with chicken entry and exit in the flock, common poultry diseases and their impacts on morbidity and mortality, seasonality of diseases, and key challenges and constraints to production in poultry systems in Ghana and Tanzania were organized into themes and analyzed using descriptive statistics. Semi-quantitative data were collected in the study, mainly from the proportional piling, scoring, and ranking exercises. These were entered into a Microsoft Access 2002 database (15) and exported to STATA 15 (16), for analysis. Data obtained from the scoring tools were summarized using means and medians to determine central tendency and range and standard deviation to assess dispersion. The seasonal calendar aspects were summarized using means and presented in tabular form.

## Results

### Characteristics of the FGD participants

In Tanzania, a total of 137 people participated in the FGDs comprising 60 men and 77 women. The average age of the FGD participants was 42 years old, with a range of 18–82 years. Most participants (69%) had attained a primary level of education while 22% had completed their secondary education. The remaining 9% of participants either had a college education or informal education. Participants residing in households that rear chickens mainly for income and food security had an average of six household members. The average flock size among participants was 26 birds, ranging from 2 to 60 birds per household. Most of the birds owned by participants were local ecotypes, reared under extensive scavenging or semi-intensive production systems with some feed supplementation from kitchen leftovers or cereal grains.

In Ghana, 123 poultry producers participated in the FGDs and comprising 69 men and 54 women. Like Tanzania, the average age of the FGD participants in Ghana was 42 years old, with a range of 19–80 years. Illiteracy level was high at the study sites in Ghana, with 60% of the participants having no formal education. The farmers mainly raised local chicken ecotypes, with flock sizes ranging from 2 to 180 birds. Most of the farmers practiced small extensive scavenging and extensive scavenging production systems with a few producers supplementing feed with grains.

### Poultry production systems and husbandry practices

The study utilized the poultry production systems classification documented in FAO (17). Generally, small scale extensive scavenging and semi-intensive poultry production systems were commonly practiced at the sites in Ghana. The participants were given 100 counters to distribute based on existing poultry productions systems practiced in their communities. In the Northern region, 100% of the counters were placed under the semi-intensive system showing its dominance in the communities. In the Upper East region, an average of 90.4% of the counters from the four FGDs were placed under the free-range extensive scavenging systems (Table 2).

**Table 2:** Poultry production systems in Ghana.

Northern region		
Poultry production system (day)	Semi-intensive	
Score	100	
Type of poultry	Local chicken, Guinea fowl, Ducks, Turkeys, and Pigeons,	
Poultry breed reared	Local breed	
Reason for system practice	To improve health of birds To get birds used to the house and for identification purposes Droppings of birds used to fertilize crop fields To protect birds from going astray, predators, being stolen Protection from theft, predators, and rain and harsh weather conditions (shelter)	
Seasonality of the system	All year round. However, some farmers rear their poultry on their farms (crop fields) during the wet season	
Type of housing	Majority are mud housing roofed with thatch; few are built with Zana mats (woven grasses) roofed with thatch (grasses)	
Upper East region		
Poultry production system	Free range-extensive	Semi-intensive
Score	90.4	9.6
Type of poultry	Local indigenous, ducks, and guinea fowls	Local indigenous and guinea fowls
Poultry breed reared	Local indigenous, guinea fowls, and ducks	Local indigenous and guinea fowls
Reason for system practice	Cheaper to practice	Keeps poultry from straying
Seasonality of the system	All year round	Only at the younger stage of poultry
Type of housing (day)	Free range extensive	Backyard extensive
Type of housing (night)	Local hen coops made of mud and roofed with thatch	Local hen coops made of mud and roofed with thatch, Local hen coops made of mud, and roofed with thatch in a fenced area

Producers indicated a preference for the extensive scavenging system due to minimal inputs, particularly feed-related costs. In the semi-intensive system, the birds roam in a confined space during the day and are provided supplemental feed consisting of kitchen leftovers and occasionally grains or grain by-products. The semi-intensive system enables collection of chicken manure for use as crop fertilizer. In the extensive scavenging system, birds are allowed to roam freely without confinement and may or may not be provided supplemental feed. The types of birds kept under these two systems included the local chicken ecotypes, guinea fowl, ducks, turkeys, and pigeons (Table 2).

Similarly, small-scale extensive scavenging and semi-intensive systems were commonly practiced in the Singida and Dodoma regions of Tanzania, estimated at 52 and 42%, respectively (Table 3). The main poultry types reared under these systems included local chicken ecotypes, quails, turkeys, and ducks. In Kongwa DC in the Dodoma region, small-scale intensive poultry systems were practiced by a few farmers (6%). This involved confinement of birds within a fenced area and in chicken houses (coops) with commercial feed rations. In such systems, some level of biosecurity was practiced. Poultry types reared under this system included improved dual-purpose chickens (Sasso), exotic breeds, and local chicken ecotypes.

**Table 3:** Poultry production systems in Tanzania.

Poultry production system	Free range extensive (in both regions)	Semi-intensive (in both regions)	Intensive/Total confinement (practiced mainly in Kongwa DC)
Score (%)	51.6	42.2	6.2
Type of poultry	Chickens, ducks and guinea fowl, quail, and turkey	Chickens, ducks and guinea fowl, quail, and turkey	Chickens, ducks, guinea fowl, turkeys, quail, and pigeon
Poultry breed reared	Local indigenous, dual purpose	Local indigenous, Exotic, dual purpose	Local indigenous, Exotic, dual purpose
Reason for system practiced	Reduce housing cost, reduce cost of feeding, poverty	Avoid destruction of crop, biosecurity, security against theft and predators, reduce the possibility of contracting disease from other flocks, and prevent breeds mixing (undesirable traits) from other flocks	Security reasons, easy entry, and disease management
Seasonality	All year around	All year around	All year around
Housing day		Poultry shades (Kibaigwa-Kongwa DC)	Poultry shades (half bricks, half wire mesh)
Housing night	Kitchen and muddy huts, no house special for chicken (most of the communities live with their birds in house), Poultry shades/house depending on owner’s economy	Muddy huts, no house special for chickens, Poultry shades/house depends on the owner’s economy	Poultry shades (half bricks, half wire mesh)

Participants indicated the poultry husbandry practices implemented in their communities. For each practice, the number of participants implementing the practice was taken through a handcount. In both countries, the common poultry husbandry practices included vaccination, preventive treatments for infectious diseases (e.g., deworming, use of herbs, and antibiotics such as amoxillin), curative treatment for diseases, feeding and watering, and ensuring proper sanitation and cleanliness of poultry houses (coops), especially under the semi-intensive system (Tables 4, 5). Selection of cocks was common in both regions in Ghana and de-beaking was common in the Upper East region (Table 4). In Tanzania, brooding of chicks was common in Ulemo site in Singida region (Table 5). These tasks were shared by all family members. Treatment and vaccination practices were performed by family members, veterinarians, and livestock and veterinary extension workers. In Tanzania, herbalists were also found to participate in the treatment of poultry.

**Table 4:** Poultry husbandry practices in Ghana—participant handcount.

Poultry husbandry practices	Northern region				Upper East region			
	Kumbungu (*n* = 15)	Gbulung (*n* = 15)	Diare (*n* = 15)	Savelugu (*n* = 15)	Nyariga (*n* = 15)	Sherigu (*n* = 15)	Zebila (*n* = 15)	Kukore (*n* = 17)
Vaccination (NCD)	15	8	10	11	8	9	8	10
Deworming	6	3	11	7	5	9	7	6
Mineral supplementation	0	0	0	0	3	3	2	3
Watering	15	15	15	15	15	15	15	17
Selection of cocks for breeding	15	11	0	1	12	14	12	10
De-beaking	0	0	0	0	10	6	10	8

**Table 5:** Poultry husbandry practices in Tanzania—participant handcount.

Poultry husbandry practices	Dodoma				Singida			
	Mbande/Sejeli (*n* = 17)	Kibaigwa (*n* = 17)	Gwandi (*n* = 17)	Farkwa (*n* = 17)	Old Kiomboi (*n* = 17)	Ulemo (*n* = 18)	Ikhanoda (*n* = 17)	Mtinko (*n* = 15)
Vaccination (NCD)	9	12	12	0	12	15	11	4
Chicken coop and maintaining cleanliness	11	12	15	14	15	4	8	11
Watering	14	8	13	14	2	0	10	11
Treatment	9	12	13	4	15	0	2	0
Brooding chicks^*^	0	0	0	0	3	15	0	0
Supplementation	0	0	0	0	0	11	0	0
De-beaking	0	7	0	0	0	0	0	0
Mineral supplementation	0	3	0	0	0	0	0	0
Biosecurity measures (dusting against fleas)	0	0	0	14	0	0	0	0

* Brooding chicks—hen and her chicks are separated from the flock and reared in an enclosure until chicks attain 2 months of age.

### Main constraints to poultry husbandry and management

The constraints to proper poultry flock husbandry and management faced by smallholder poultry farmers were similar in Tanzania and Ghana. Common constraints included limited financial resources and capital, limited access to critical inputs, such as vaccines and agricultural and veterinary extension services, lack of knowledge and training in poultry husbandry, shortage of quality feed, and disease threats. The study revealed other challenges, including extreme weather events impacting feed availability, and limited decision-making power of women in some areas, particularly in Northern region in Ghana on how financial resources stemming from poultry sales were utilized in the household.

### Chicken flock dynamics

#### Chicken flock entry and exit

In both countries, the main source of chicken entry into the flocks was through hatching, accounting for 70–88% of entries in both countries (Table 6). Another source of entry was through purchases, especially at the Ghana sites, accounting for 13–18% of all entries compared to 7–9% in Tanzania. Entries through gifts, exchanges, and borrowing were minimal.

**Table 6:** Exit and entry of chickens in producers’ flocks.

Entry/exit	Ghana		Tanzania	
	Northern (*n* = 4)	Upper East (*n* = 4)	Singida (*n* = 4)	Dodoma (*n* = 4)
*Sources of entry (average %)*				
Purchase	17.5	12.8	8.8	7.0
Gifts	7.3	0	3.0	2.5
Hatching	69.5	87.3	82.5	87.5
Exchange	5.8	0	4.5	3.0
Borrowing	0	0	1.3	0.0
*Sources of exit (average %)*				
Sales	20.0	19.8	22.5	45.0
Consumption	7.3	17.5	10.5	12.8
Gifts	5.5	5.3	4.5	2.0
Theft	3.8	6.8	2.0	4.5
Trading for services	0.0	0.0	3.0	0.0
Death	23.5	38.5	35.0	14.5
Disease	15.0	27.8	23.3	10.7
Accident	4.3	4.0	3.0	1.3
Predators	4.3	6.8	8.8	5.3

The main factors associated with chickens exiting the flocks in both countries were deaths and sales. Sales accounted for about 20% of poultry exits in both regions in Ghana. In Tanzania, sales contributed to 23 and 45% of exits in Singida and Dodoma regions, respectively. In Ghana, deaths accounted for 24 and 39% of the exits in Northern and Upper East regions, respectively. In Tanzania, deaths accounted for 35 and 15% of exits in Singida and Dodoma, respectively. The main cause of death, as reported by participants, was disease, accounting for 11–28% of the mortality. Other causes of death included traumatic injuries and predation (Table 6). Home consumption was also a source of flock exits accounting for 7–18% of exits. Other factors included gifting, theft, and trading of birds for services, though these contributed to only 3–7% of flock exits.

### Poultry diseases

#### Common poultry diseases and seasonal occurrence

The FGD participants were asked to indicate the most common poultry diseases affecting chickens in their area. They then prioritized the diseases based on commonality in terms of occurrence using pairwise ranking to obtain the top five diseases. Table 7 shows the priority diseases identified in Ghana. Fowl pox and NCD were prioritized in all the eight FGDs conducted in Ghana in both regions. In the Upper East region, NCD had the highest overall score in terms of the cause of morbidity, followed by fowl pox.

**Table 7:** Common poultry diseases in Ghana.

Disease/Region	Local name	No. of FGDs indicating disease	Average score in terms of morbidity
*Upper East*			
Newcastle disease	“Nokum”	4	49.3
Fowl pox	“Kanzeriba”	4	13.8
Coccidiosis	“Binsabla”	2	4.5
Botulism	“Nofaam”	2	5.0
Chronic respiratory disease	“Fewufewu”	2	5.5
Bacillary white diarrhea	“wmefobe”	1	3.5
*Northern*			
Newcastle disease	“Vilovilo”	4	3.0
Fowl pox	“Chara”	4	5.3
Chronic respiratory disease	-	1	2.8
Worm infestation	“Gara”	4	12.5
Chemical poisoning	“Cherigili”	2	11.3
Coryza	“Nimbihi Kpula”	2	2.8
Indigestion	“Fegala”	1	2.5
Fowl lice	“Noyun”	1	2.5

Other diseases that were prioritized included coccidiosis, chronic respiratory disease of unknown etiology, and botulism. In the Northern region, the diseases/conditions that had the highest score in terms of causes of morbidity were worm infestation and what producers indicated was chemical poisoning. NCD and fowl pox, although mentioned by all four FGDs in the Northern region, had a relatively lower score in terms of causes of morbidity. Worm infestation was mentioned by all four FGDs in Northern region. Chemical poisoning was mentioned by two FGDs and was scored highly as a cause of morbidity. The clinical symptoms included severe depression, loss of feathers, inability of birds to stand, and rapid death. These symptoms, according to the producers’ perceptions were the result of chemical poisoning in the flock. It is important to note here that the participant’s ranks were often based on perceptions rather than a definitive diagnosis.

Table 8 shows the prioritized diseases in Tanzania. In both Dodoma and Singida, NCD was identified in all four FGDs held per region and had the highest scores in terms of the cause of morbidity. Fowl pox, coryza, and coccidiosis were prioritized by at least two of the four FGDs. Other diseases of priority included collibacilosis, fowl typhoid, and worm infestation.

**Table 8:** Common poultry diseases in Tanzania.

Disease/Region	Local name	No. of FGDs indicating disease	Average score in terms of morbidity
*Singida*			
Newcastle disease	“Kideri”	4	17.5
Coryza	“Mafua”	3	3.0
Fowl pox	“Ndui”	3	3.5
Coccidiosis	“Kuhara damu”	2	2.8
Collibacilosis	“Ini kubwa”	1	1.3
Vitamin and mineral deficiency	“Kuvimba macho”	1	2.0
*Dodoma*			
Newcastle disease	“Kideri”	4	23.8
Coryza	“Mafua”	2	3.0
Fowl pox	“Ndui”	2	2.0
Fowl typhoid	“Taifodi/kuhara chokaa”	1	1.8
Encephalomyelitis	-	1	1.3
Vitamin and mineral deficiency	“Kuvimba macho”	1	0.5
Coccidiosis	“Kuhara damu”	2	3.3
Colibacillosis	“Ini kubwa”	1	1.3
Worm infestation	“Minyoo”	1	0.8

The monthly occurrence of NCD, the major disease of importance, differed between Tanzania and Ghana, however, the seasonal pattern of the disease was similar in that NCD was most prevalent during the dry seasons in each country (Table 9). In Ghana, study participants reported that NCD was most prevalent in the months of January to April and November to December. In the Northern region of Ghana, worm infestation was reported to occur year-round. However, most of the disease was reported in the months of June, November, and December in a given calendar year by three of the four FGDs. In both regions of Ghana, fowl pox was common between June and December.

**Table 9:** Seasonal occurrences of chicken diseases in Ghana.

Region/Disease	Calendar months											
	J	F	M	A	M	J	J	A	S	O	N	D
*Northern*												
Chemical poisoning			X	XXX	XX	X	XXXX	X				
Coryza	XX	X						X	XXX	X	X	XXX
Chronic respiratory disease	X	X									XXX	XX
Fowl lice					X	XX	XX	X	X			
Fowl pox						X	XXX	XX	X		XXX	XX
Indigestion												
Newcastle disease	XX	X	XXXX	XX							X	XXX
Worm infestation	X	X	X	X	XX	XX	XX	XX	X	X	XX	X
*Upper East*												
Newcastle disease	XX	XX	X	X							X	XX
Fowl Pox							XX	XXX	XXX	XX	XX	X
Coccidiosis						XX	XXX	X	X	X	XXX	XXX
Chronic Respiratory Disease	X	X	X							X	XX	XX
Botulism	XX	XXX	X	XX	XX	X						XX
Bacillary white diarrhea	XX	XXX	XX	XX	XX	X						XX
Others			X	XX	XX	X						

X, Number of FGDs reporting the disease in the calendar month.

In Tanzania, the participants from the four FGDs reported that NCD was more prevalent between the months of May and December in a given calendar year. In the Singida region of Tanzania, fowl pox and colibacillosis were reported to occur in poultry year-round. In Dodoma, infectious coryza, fowl typhoid, fowl pox, and coccidiosis were also reported to occur in poultry throughout the calendar year (Table 10).

**Table 10:** Seasonal occurrences of chicken diseases in Tanzania.

Region/Disease	Calendar months											
	J	F	M	A	M	J	J	A	S	O	N	D
*Singida*												
Worm infestation	X	XX	XX	X	X							X
Newcastle disease						X	X	X	XX	XX	X	X
Infectious Coryza					X	XX	XX	X	X	XX	X	X
Infectious Bronchitis					X	X	X	X	X	X	X	X
Fowl typhoid						XXX	XX	X				
Fowl pox	XX	XX	X	X	X	X	X	X	XX	X	X	X
Colibacillosis	X	X	X	X	X	X	X	X	X	X	X	X
Coccidiosis						X	X	X	X	XX	XX	XX
*Dodoma*												
Worm infestation	X	X	X	X	X							X
Vit A deficiency							X	XX	XX	X	X	
Newcastle disease	X				X	X	XX	X	X	X	X	X
Infectious Coryza	X	X	X	X	X	X	X	XX	X	X	X	X
Fowl typhoid	X	X	X	X	X	X	X	X	X	X	X	X
Fowl pox	X	X	X	X	X	X	X	XX	XX	XX	X	X
Coccidiosis	X	X	X	X	XX	XX	X	X	X	X	X	X

X, Number of FGDs reporting the disease in the calendar month.

#### Morbidity, mortality, and case fatality ratio from chicken diseases

In Tanzania, NCD was the leading cause of morbidity and mortality from disease. Mortality rates associated with NCD ranged between 21.3 and 23.3% in the Dodoma and Singida regions, respectively (Table 11). Other diseases associated with relatively high mortality in Tanzania were fowl pox, coccidiosis and colibacillosis at about 3.8–5.0%. The case fatality ratio for NCD was 0.98, showing the severity of the disease for infected birds.

**Table 11:** Morbidity and mortality rates due to poultry diseases in Tanzania.

Disease	Region					
	Singida			Dodoma		
	Morbidity rate (%)	Mortality rate (%)	Case fatality ratio	Morbidity rate (%)	Mortality rate (%)	Case fatality ratio
Newcastle (NCD)	23.3	22.8	0.98	22.5	21.3	0.95
Fowl pox	6.0	3.8	0.63	3.3	1.5	0.45
Worm infestation	0.0	0.0	0.00	0.8	0.3	0.38
Coryza	3.0	2.0	0.67	3.0	1.8	0.60
Coccidiosis	6.5	4.5	0.69	3.8	2.8	0.74
Fowl typhoid	0.0	0.0	0.00	2.5	1.3	0.52
Colibacillosis	5.0	5.0	1.00	1.3	1.3	1.00
Bronchitis	2.5	1.3	0.52	0.0	0.0	0.00
Encephalomyelitis	0.0	0.0	0.00	1.3	1.3	1.00
Vitamin A deficiency	0.0	0.0	0.00	0.5	0.3	0.60

In Ghana, NCD was the main cause of morbidity and mortality from disease in the Upper East region (Table 12). The morbidity and mortality rates were 49.3 and 40.5%, respectively with a case fatality ratio of 0.82. In the Northern region, worm infestation and chemical poisoning were indicated to be the main causes of morbidity from disease at a rate of 12.5 and 11.3%, respectively and mortality rates of 10.0 and 11.3% from disease with a case fatality ratio of 0.8–1.0.

**Table 12:** Morbidity and mortality rates due to poultry diseases in Ghana.

Disease	Region					
	Northern			Upper East		
	Morbidity rate (%)	Mortality rate (%)	Case fatality ratio	Morbidity rate (%)	Mortality rate (%)	Case fatality ratio
Newcastle (NCD)	3.0	0.3	0.10	49.3	40.5	0.82
Fowl pox	5.3	2.5	0.47	13.8	7.0	0.51
Worm infestation	12.5	10.0	0.80	0.5	0.3	0.60
Chronic respiratory disease	2.8	1.5	0.54	5.5	3.5	0.64
Chemical poisoning	11.3	11.3	1.00	0.0	0.0	0.00
Coryza	2.8	0.8	0.29	0.0	0.0	0.00
Indigestion	2.5	1.8	0.72	0.0	0.0	0.00
Fowl lice	2.5	1.0	0.40	0.0	0.0	0.00
Botulism	0.0	0.0	0.00	5.0	3.0	0.60
Bacillary white diarrhea	0.0	0.0	0.00	3.8	3.0	0.79
Coccidiosis	0.0	0.0	0.00	4.5	1.8	0.40

The morbidity rate due to NCD was low, at 3.0% in the Northern region with a mortality rate of 0.3%. In the Upper East region, fowl pox was also a cause of morbidity in chicken with a morbidity rate of 13.8%, though the mortality rate was 7.0%. Diseases such as bacillary white diarrhea and chronic respiratory disease had low morbidity rates, but relatively high case fatality ratios, indicating that infections most often result in death. Morbidity and mortality rates associated with disease in the Northern region of Ghana were relatively low, highlighting the importance of other non-disease related factors contributing to mortality in this region.

## Discussion

Small-scale semi-intensive and extensive scavenging poultry production systems were commonly practiced in the study areas. The chicken reared were mostly the local or indigenous ecotypes. The extensive scavenging system requires minimal investments with birds mainly scavenging for food and housing, if provided, is simple, made of locally available materials, and mostly provided at night for shelter (1). However, birds in these systems are vulnerable to theft, predation, and disease related losses (18). To support smallholder producers in semi-intensive and extensive systems, greater access to extension services as well as training in poultry husbandry, poultry nutrition, and biosecurity is key. As economies develop and access to inputs and markets improve, the use of improved indigenous chicken ecotypes will enable smallholders to upgrade to more market-oriented production (19), highlighting the importance of addressing challenges in the near-term to meet increasing national and international demand for animal-source protein and micronutrients for nutritional security. This also increases the bargaining power of these countries for better market access in the world agricultural market for their exports.

Hatching of eggs by broody hens or flock self-propagation was the common source of poultry flock entry among farmers at the study sites. Similarly, Kejela (20) reported hatching as a major source of flock entry in local poultry production systems in Ethiopia. A study in Kogi State in Nigeria showed that purchases from the live bird market were a major source of poultry flock entry (21). While flock self-propagation through natural incubation is a common practice among smallholders, the number of spoilt eggs may be a challenge depending on the broodiness of the hen. Besides, only a small number of eggs can be hatched at a time (22). Use of artificial incubators or hatcheries can be advantageous for producers wanting to increase the size of their flocks; given provision of optimal hatching conditions can reduce risk of egg breakage and increase hatching rates (23). Hatcheries can be particularly useful for producing ecotypes with desired traits through genetic selection, such as enhanced resistance to NCD in systems where the disease is prevalent (24). For instance, in Tanzania, there are several small hatcheries supplying day old chicks of indigenous chicken ecotypes and specialized dual-purpose breeds to the semi-intensive production systems in the peri-urban and urban areas (8). The hatcheries obtain the fertilized eggs from breeder farms. In Northern Ghana, there are very few incubator businesses due to unreliable electricity supply especially in rural locations. Various models of mini hatcheries constructed from cheap available local materials such as rice husk, quilts, and sand, to retain heat have been tested in Asia, with opportunities for scaling in sub–Saharan Africa. For instance, IFAD (25) shows estimates of 80–85% hatching rates from sand-based mini hatcheries.

Infectious poultry diseases are among the key factors driving low productivity of village poultry production systems around the world. These systems face several constraints, but diseases are highly ranked as the primary factor for birds exiting flocks in small-scale extensive scavenging and semi-intensive systems (26). In this study, NCD was ranked as the highest cause of morbidity and mortality in chickens in the two countries. The disease occurred mainly during the calendar months coinciding with the dry season in both countries. Studies show that seasonality of outbreaks is influenced by changes in climatic conditions leading to stress, which predisposes birds to the disease, and hot, dry and windy periods, which encourage airborne spread of the virus (27). Several studies in sub-Saharan Africa settings have shown the substantial losses due to NCD in village poultry systems (28–30) resulting from high mortalities (11). Dankwa et al. (31) in their study of Northern Ghana show recurrent outbreaks of NCD in November and January and resulting losses of flocks. The findings confirm that NCD is endemic in most rural extensive and semi-intensive systems in sub-Saharan Africa where access to vaccination and extension services is limited, and reliable cold chain is lacking (24). Although NCD was the leading cause of morbidity in chickens, efforts to control other diseases particularly fowl pox, coccidiosis and infectious coryza should be enhanced. Several studies such as Awuni (32) and Chota et al. (33) have found fowl pox and coccidiosis as major causes of chicken mortalities in extensive scavenging systems in Northern Ghana and Central Tanzania.

The poultry diseases were often not definitively diagnosed. In Northern region in Ghana for instance, farmers perceived high mortality in birds to result from chemical poisoning. However, there was no proper diagnosis from the veterinarians, possibly due to shortage of veterinary service providers and lack of laboratory diagnostic facilities (13). Besides, the high flock morbidity due to worm infestation in Northern region may be the result of poor access to veterinary services as well as lack of knowledge in appropriate husbandry practices, indicated as key constraints by the poultry farmers. These challenges point to the need for increased investments and support by both public and private sector in both countries in poultry disease control and prevention programs targeted particularly to rural areas.

Poor access to veterinary services and quality veterinary products coupled with lack of knowledge in appropriate husbandry practices, influence producers to use unorthodox disease preventive measures. In Tanzania for instance, herbalists were engaged in treatment of poultry using locally available herbs. In Northern region of Ghana, poultry producers indicated use of local herbs and antibiotics such as amoxillin for disease prevention (8). Okitoi et al. (34) reported similar findings in Kenya. In rural settings, these may be the only options that households have, due to limited access to veterinary service providers and poor access to veterinary products. Use of antibiotics in chicken flock without proper prescription may result in public health concerns as the chicken products and by-products enter the food chain. Studies such as Enahoro et al. (13) report poor access to veterinary products in rural locations in Northern Ghana and Central Tanzania due to long distances and supply shortages resulting in high cost of the products.

## Study limitation

Although this study provides useful information on disease and health challenges in the study sites, it is based on qualitative inquiry and poultry producers’ perspectives. The diseases identified are based on the symptoms and farmer experiences rather than confirmatory tests through laboratory diagnostics. Such studies can be complemented with quantitative studies that also utilize disease diagnostics.

## Conclusion

Smallholder producers still dominate the poultry sector in the two countries with extensive scavenging and semi-intensive as the dominant production systems. These farmers face high burden of poultry diseases, especially NCD, limited access to critical health inputs, such as vaccines, veterinary and extension services, and lack of knowledge in appropriate poultry husbandry practices. The high proportion of flock morbidity and mortality due to NCD, coupled with poor access to veterinary inputs in such systems calls for concerted efforts and multiple throng approaches to control the disease. Tapping into the diverse genetic reservoir of chicken ecotypes with enhanced resistance to NCD coupled with models for enhancing vaccination supply and use are potential avenues to address the NCD constraint.

Several of the poultry health constraints require both public and private sector interventions to strengthen the village poultry systems. Besides, improvement of village chicken production will require a holistic approach focusing on both husbandry practices as well as disease control especially strategic control programs of common diseases such as NCD, and fowl pox. Such interventions include investments in expansion of veterinary and agricultural extension services in rural areas diagnostic facilities, and enhanced capacity of producers in poultry husbandry and disease prevention measures.

## Data availability statement

The raw data supporting the conclusions of this article will be made available by the authors, without undue reservation.

## Ethics statement

The studies involving human participants were reviewed and approved by Ethics Committee for Basic and Applied Sciences (ECBAS), University of Ghana, Ref. No. ECBAS046/18-19. Ethical clearance was obtained in Tanzania from the Office of the Vice-Chancellor, Sokoine University of Agriculture, and research permits for Singida (Ref. No. DPRTC/R/142/Vol. I /42) and Dodoma (Ref. No. DPRTC/R/142/Vol. I/43). Informed written consents were obtained from all participants in the study. The patients/participants provided their written informed consent to participate in this study.

## Author contributions

EAO, DE, TK, and HZ conceived the study. MD and EAO developed the methodologies and tools. GC and YA supervised the field data collection with oversight from EAO and DE. CK conducted the analysis and wrote the initial draft of the manuscript. TK, DE, PM, BBK, and HZ provided comments on the draft, and EAO synthesized input from all co-authors into the final draft. All authors contributed to the article and approved the submitted version. EAO, CK, MD, TK, DE, GC, YA, PM, BBK, and HZ listed have made a substantial contribution to the work and have approved this submission for publication.

## Funding

This study was funded by the US Agency for International Development (USAID) Feed the Future Innovation Lab for Genomics to Improve Poultry AID-OAA-A-13-00080. We also acknowledge funding of the CGIAR Initiative on Sustainable Animal Productivity and all donors and organizations, which globally support the work of the CGIAR Initiatives through their contributions to the CGIAR Trust Fund. Any opinions, findings, conclusions, or recommendations expressed here are those of the authors alone.

## Conflict of interest

The authors declare that the research was conducted in the absence of any commercial relationships that could be construed as a potential conflict of interest.

## Publisher’s note

All claims expressed in this article are solely those of the authors and do not necessarily represent those of their affiliated organizations, or those of the publisher, the editors and the reviewers. Any product that may be evaluated in this article, or claim that may be made by its manufacturer, is not guaranteed or endorsed by the publisher.

## Footnotes

^1^Disease resistance is in part genetically controlled, allowing for genetic improvement of targeted traits, including increased survival or resilience in the face of infection as well as a more robust host immune response to Newcastle disease virus vaccination (12).
